# The effects of non-surgical periodontal treatment on glycemic control, oxidative stress balance and quality of life in patients with type 2 diabetes: A randomized clinical trial

**DOI:** 10.1371/journal.pone.0188171

**Published:** 2017-11-16

**Authors:** Hirofumi Mizuno, Daisuke Ekuni, Takayuki Maruyama, Kota Kataoka, Toshiki Yoneda, Daiki Fukuhara, Yoshio Sugiura, Takaaki Tomofuji, Jun Wada, Manabu Morita

**Affiliations:** 1 Department of Preventive Dentistry, Okayama University Graduate School of Medicine, Dentistry and Pharmaceutical Sciences, Okayama, Japan; 2 Advanced Research Center for Oral and Craniofacial Sciences, Okayama University Dental School, Okayama, Japan; 3 Center for Innovative Clinical Medicine, Okayama University Hospital, Okayama, Japan; 4 Department of Community Oral Health, Asahi University School of Dentistry, Mizuho, Japan; 5 Department of Nephrology, Rheumatology, Endocrinology, and Metabolism, Okayama University Graduate School of Medicine, Dentistry, and Pharmaceutical Sciences, Okayama, Japan; The Ohio State University, UNITED STATES

## Abstract

**Aim:**

The purpose of this study was to investigate the effects of non-surgical periodontal treatment on hemoglobinA1c (HbA1c) levels, oxidative stress balance and quality of life (QOL) in patients with type 2 diabetes mellitus (T2DM) compared to no periodontal treatment (simple oral hygiene instructions only).

**Methods:**

The design was a 6-month, single-masked, single center, randomized clinical trial. Patients had both T2DM and chronic periodontitis. Forty participants were enrolled between April 2014 and March 2016 at the Nephrology, Diabetology and Endocrinology Department of Okayama University Hospital. The periodontal treatment group (n = 20) received non-surgical periodontal therapy, including scaling and root planing plus oral hygiene instructions, and consecutive supportive periodontal therapy at 3 and 6 months. The control group (n = 17) received only oral hygiene instructions without treatment during the experimental period. The primary study outcome was the change in HbA1c levels from baseline to 3 months. Secondary outcomes included changes in oxidative stress balance (Oxidative-INDEX), the Diabetes Therapy-Related QOL and clinical periodontal parameters from baseline to 3 months and baseline to 6 months.

**Results:**

Changes in HbA1c in the periodontal treatment group were not significantly different with those in the control group at 3 and 6 months. Systemic oxidative stress balance and QOL significantly improved in the periodontal treatment group compared to the control group at 3 months. In the subgroup analysis (moderately poor control of diabetes), the decrease in HbA1c levels in the periodontal treatment group was greater than that in the control group at 3 months but not significant.

**Conclusions:**

In T2DM patients, non-surgical periodontal treatment improved systemic oxidative stress balance and QOL, but did not decrease HbA1c levels at 3 months follow-up.

**Trial registration:**

Current Controlled Trials UMIN-ICDR UMIN 000013278 (Registered April 1, 2014).

## Introduction

Type 2 diabetes mellitus (T2DM) is a common chronic disease and can cause various health issues. Treatment goals include the prevention of complications as well as glycemic control [1]. A position statement by the Diabetes Association in 2016 [2] also suggests that a complete medical evaluation should be performed to detect diabetes complications.

Periodontitis and diabetes are chronic diseases with an established bidirectional relationship. Periodontitis is recognized as the sixth complication of diabetes [3] and is a major cause of tooth loss in adults [4]. Recent reviews suggest that periodontitis adversely affects diabetes outcomes. The effect of periodontitis on diabetes may be explained by the increase in levels of systemic proinflammatory mediators, which exacerbates insulin resistance [5]. However, evidence for treatment benefits remains controversial due to the variety of the sample population, methods and control of T2DM [2, 6–14].

Recent studies suggest that oxidative stress plays an important role in the pathophysiology of both T2DM and periodontitis [15–25]. We previously showed that non-surgical periodontal treatment (scaling and root planing and supportive periodontal therapy) reduced systemic oxidative stress in healthy patients [26]. Thus, the periodontal treatment may also improve oxidative stress in T2DM patients.

The complications of diabetes significantly impair quality of life (QOL) [27–31], and the reduction in QOL may interfere with diabetes treatment. Thus, improvement of these complications may contribute to better QOL for diabetes patients. Although recent reviews suggest that outcome measures in clinical trials should include QOL, there are no studies focusing on the effects of periodontal treatment on QOL [9, 32]. Therefore, we hypothesized that non-surgical periodontal treatment reduces systemic oxidative stress and HbA1c levels as well as improves QOL in T2DM patients. The purpose of this randomized clinical trial was to investigate the effects of non-surgical periodontal treatment on HbA1c levels, oxidative stress and QOL in patients with T2DM and periodontitis compared to no periodontal treatment.

## Materials and methods

### Trial design

The trial was a single-masked, parallel-design, single center, randomized clinical trial that enrolled participants from outpatient medical and dental clinics in Japan following the CONSORT guidelines. The study protocol was approved by the Ethics Committee, Okayama University Graduate School of Medicine, Dentistry and Pharmaceutical Sciences and Okayama University Hospital (no. d11004). Physicians enrolled the participants during the diabetes care visits. All patients provided written informed consent. An independent center reviewed the safety data throughout the trial. Code breaking was performed after the final statistical analysis. Before and after the trial, there were no changes in eligibility criteria and outcomes.

### Randomization

Randomization was stratified by levels of HbA1c (< 8% vs. ≥ 8%), insulin (use vs. no use) and the number of medications (≤ 2 vs. >2). Each selected patient received a code number and one of the study coordinators (T.M.) used a computer-generated table to randomly allocate patients to one of the two groups (control and periodontal treatment group as below) (allocation ratio; 1:1) [33].

### Blinding

Study personnel, including the periodontal examiners, laboratory personnel who performed the HbA1c analyses and the investigator responsible for the data analysis were blinded to the treatment assignment.

### Participants

Participants were recruited between April 2014 and March 2016 at the Nephrology, Diabetology and Endocrinology Department of Okayama University Hospital. The inclusion criteria (see below) were selected to maximize the chance that a participant would complete the 6-month trial. Men and women (≥ 30 years) were eligible if they had physician-diagnosed T2DM (diagnosed at least 2 months prior to the study), reported that they were able to make hospital visits throughout the trial, were in the care of a physician for their diabetes, and agreed to not change their diabetes medications during the trial unless medically indicated. Patients also required a diagnosis of mild to advanced chronic periodontitis, defined as ≥ 2 interproximal sites with clinical attachment level (CAL) ≥ 3 mm and ≥ 2 interproximal sites with probing pocket depth (PPD) ≥ 4 mm (not on the same tooth) or one site with PPD ≥ 5 mm [34]. Radiographs were used to confirm a diagnosis of chronic periodontitis [35]. Exclusion criteria included pregnancy, inappropriate status for the trials, such as limited life expectancy and diabetes-related emergency, and receiving periodontal treatment in the prior 6 months [35].

### Intervention

Initially, all participants received one session of supra-gingival plaque removal and oral hygiene instructions. The patients of the periodontal group were treated by a protocol adapted from the Japanese Society of Periodontology. The non-surgical periodontal treatment consisted of at least 60 minutes of scaling and root planing using curettes (BioGent Curette, Hu-Friedy, Chicago, IL, USA; Scaler YB, YDM Corporation, Saitama, Japan) and an ultrasonic instrument (Varios 750, NSK, Tochigi, Japan) during 2 or more sessions completed within 42 days after the baseline visit [36]. Completeness of therapy was assessed by the study therapists and confirmed by a study dentist. During the treatment, the therapists provided oral hygiene instructions, professional toothbrushing and mechanical tooth cleaning. Three and 6 months after the baseline visit, participants in the treatment group received periodontal maintenance therapy including oral hygiene instructions and oral prophylaxis for approximately 1 hour during a single session. Participants in the control group received only oral hygiene instructions at the baseline and 3 and 6 month visits. After the study completion, the control group participants received scaling and root planing.

### Outcome assessment

The primary study outcome was the change in HbA1c levels from baseline to 3 months. Secondary outcomes included changes in oxidative stress balance, glycated albumin, QOL and clinical periodontal parameters from baseline to 3 months and baseline to 6 months. Change in diabetes medications at 3 and 6 months, and the need for periodontal rescue therapy and diabetes rescue therapy were evaluated as safety outcomes. A change in medication was defined as more than 2-fold change in dosage for a hyperglycemic drug, more than 10% change in dosage for insulin, or change in an oral hyperglycemic agent or insulin [35].

### Sample size calculation

Sample size was estimated assuming a 0.8% (SD, 0.8%) or greater reduction in HbA1c levels from baseline to 3 months in the periodontal treatment group compared with the control group [37]. Based on the data, it was determined that 17 subjects per group would be necessary to provide an 80% power with an alpha of 0.05, 2-tailed and unpaired *t* test. Assuming an attrition rate of 15% [38], the planned sample size was 40 participants (20 in each treatment group).

### Data collection

Data were collected by trained and certified study personnel. Study personnel recorded medical history, medication use, demographics, and lifestyle information including sex, age, height, weight, drinking habit, smoking habit, frequency of toothbrushing, use of interdental brush and also administered the Diabetes Therapy-Related QOL (DTR-QOL) questionnaire [39]. The DTR-QOL includes 4 factors: "burden on social activities and daily activities" (factor 1), "anxiety and dissatisfaction with treatment" (factor 2), "hypoglycemia" (factor 3), and "satisfaction with treatment" (factor 4) [39].

### Oral examination

The periodontal examiners (dentists) performed the following oral examination. PPD and CAL were determined at six sites (mesio-buccal, mid-buccal, disto-buccal, mesio-lingual, mid-lingual and disto-lingual) on all teeth using a color-coded probe (CP-11 Color-Coded Probe, Hu-Friedy). Sites that bled upon gentle probing (25 g probing force) were recorded, and the proportion of sites with bleeding on probing (BOP) and the number of BOP-positive teeth were measured in each subject. Plaque Control Record was measured after erythrosine staining, and recorded with respect to their relative location to the gingival margin at four sites (mesial, distal, buccal and lingual) around each tooth [40]. All clinical procedures were performed by two calibrated dentists. To check the intra- and inter-examiner agreement, measurements of PPD and CAL were recorded and repeated within a 2-week interval in two randomly selected chronic periodontitis patients. Data were analyzed with the non-parametric κ test and intra-class correlation was determined. The κ coefficients for intra- and inter-examiner and intra-class correlation coefficients were >0.8.

### Laboratory measures

At the clinical laboratory of Okayama University Hospital, venous blood samples were collected prior to periodontal measures or therapy. Fresh whole-blood samples were refrigerated and HbA1c levels were analyzed by high-performance liquid chromatography (Tosoh HLC-723G8, glycated hemoglobin analyzer, Tosoh Medics Inc., Tokyo, Japan). Serum samples were snap frozen and shipped on dry ice for analysis of levels of glycated albumin, lipids and creatinine by enzymatic methods using the automatic analyzer (JEOL BM 8040 and JCA-BM 6050, JEOL Ltd., Tokyo, Japan). High sensitive CRP was measured by an ELISA kit (High Sensitive Enzyme-Linked Immunosorbent Assay (ELISA) Kit for CRP, Cloud-Clone Corp., Houston, TX, USA). Levels of serum reactive oxygen metabolites (ROMs) (whole oxidant capacity of serum against N, N-diethylparaphenylendiamine in acidic buffer) were measured using a spectrophotometer (Diacron International, Grosseto, Italy), as reported previously [41]. Briefly, 20 μL serum samples were gently mixed with 1.2 mL acetate buffer (pH 4.8) in a cuvette, and 20 μL of chromogenic substrate (N, N-diethylparaphenylendiamine) was added. The cuvette was immediately incubated in the thermostatic block of the analyzer for 5 minutes at 37°C. Absorbance at 505 nm was automatically recorded. The Carratelli unit (CARR U), where one CARR U corresponds to 0.08 mg/dL hydrogen peroxide, was used as the measurement unit. In order to determine total serum anti-oxidant capacity, data from the OXY-adsorbent test were collected using a spectrophotometer (Diacron International) [42]. This test evaluates the capacity of serum to oppose the massive oxidative action of a hypochlorous acid (HClO) solution. Briefly, 10 μL of standards or samples, previously diluted 1:100 with distilled water, were added and mixed with 1 mL of the HClO solution. After 10-min incubation at 37°C, 10 μL of chromogenic mixture (solution provided in the kit) was added. Absorbance at 505 nm was measured immediately by the spectrophotometer. Total anti-oxidant capacity was expressed as micromoles of HClO consumed by 1 mL of sample (μmol HClO/mL). Furthermore, standardized values of the ROM and OXY-adsorbent tests were used to represent the Oxidative-INDEX as oxidative stress balance [43]. The Oxidative-INDEX was calculated by subtracting the OXY standardized variable from the ROM standardized variable. High Oxidative-INDEX values indicate high oxidative stress in the blood.

### Adverse events and safety monitoring

Oral symptoms were recorded 2 weeks following periodontal treatment (periodontal treatment group) or baseline for the control group. There were no cases in which rescue therapy was administered to patients with acute changes in periodontitis. After the trial, patients were referred for follow-up periodontal care or additional treatment as needed.

### Statistical analysis

Data analysis was performed using the Statistical Package for the Social Sciences (SPSS version 19, IBM, Tokyo, Japan). Data were summarized using means (± SDs) for continuous variables and frequencies (percentages) for categorical variables. We used an analysis of covariance (ANCOVA) for primary outcome and for secondary outcomes using the intention-to-treat analysis. The ANCOVA model included use of insulin, number of diabetes medication and HbA1c as covariate. Adjusted differences and 95% confidence intervals (CIs) were determined. P values were based on t-tests comparing mean changes between the control and periodontitis groups with Bonferroni’s method from ANCOVA. Missing data were managed according to the last observation carried forward analysis in accordance with the guidelines [44].

A per-protocol analysis based on data available both at baseline and the 3/6-month visits was also performed without imputation. Subgroup analyses were preplanned for HbA1c levels. In the subgroup analysis, we focused on moderately poor glycemic control (HbA1c; 7.0 to 10.0%) [35, 45]. To check the attrition bias, we compared the baseline characteristics between retained and dropout groups using chi-squared test and t-test. The significance level was p < 0.05.

## Results

### Retention of participants

**Fig 1:**
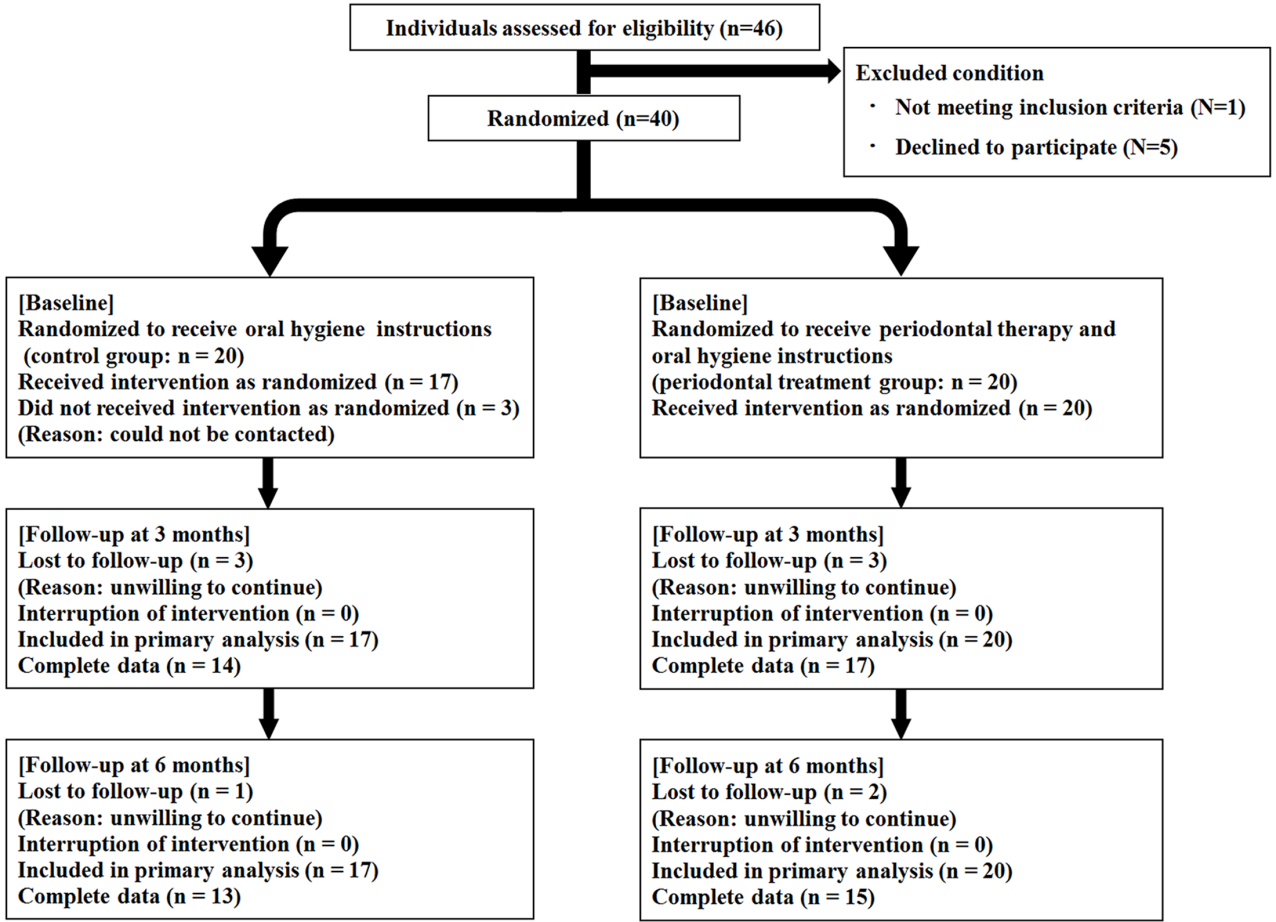
Flowchart of the study design.

As shown in Fig 1, 46 individuals were screened and 40 were randomized between April 2014 and March 2016. When the number of participants reached 40, the recruitment was ended.

Of the 40 randomized participants, 37 (92.5%) were included in the primary analysis, and 28 (70.0%) completed the study; retention in the periodontal treatment group was 15/20 and 13/20 in the control group. Baseline characteristics were similar between the two groups (Table 1).

**Table 1:** Characteristics of participants at baseline.

Parameter		Control group (N = 17)	Periodontal treatment group (N = 20)
Sex	Male	15 (88.2)*	13 (65.0)
Age (years)		62.8 ± 12.1^†^	61.2 ± 9.2
Use of insulin		8 (47.1)	6 (30.0)
Medical history except for diabetes (number)	> 2	17 (100.0)	18 (90.0)
Medication except for diabetes (number)	> 2	17 (100.0)	19 (95.0)
BMI (kg/m^2^)		27.0 ± 4.4	25.4 ± 3.6
Exercise time (hour/week)		1.3 ± 2.7	2.3 ± 2.3
Smoker (person)		5 (29.4)	2 (10.0)
Drinker (person)		7 (41.2)	7 (35.0)
Frequency of toothbrushing (times/day)	≥ two times	7 (41.2)	12 (60.0)
Use of interdental brush (number)		4 (23.5)	5 (25.0)
HbA1c (%)		7.7 ± 1.2	7.5 ± 1.7
Glycated albumin (mg/dL)		19.3 ± 3.4	19.3 ± 4.7
Cr (mg/dL)		1.0 ± 0.5	0.9 ± 0.3
TG (mg/dL)		171.3 ± 91.1	165.6 ± 110.4
HDL-C (mg/dL)		49.5 ± 13.3	55.9 ± 11.4
LDL-C (mg/dL)		98.2 ± 19.5	95.4 ± 27.2
Hs-CRP(ng/mL)		4287.0 ± 1048.3	5153.3 ± 1780.2
Oxidative INDEX		0.0 ± 1.7	0.1 ± 1.5
DTR-QOL	Factor 1	61.4 ± 22.2	72.6 ± 26.0
	Factor 2	33.7 ± 11.3	36.6 ± 10.6
	Factor 3	19.2 ± 7.3	21.5 ± 6.7
	Factor 4	19.5 ± 5.0	17.8 ± 5.6
	Total	133.8 ± 39.0	148.7 ± 39.0
Number of teeth present		24.8 ± 4.8	24.3 ± 6.2
Mean PPD (mm)		2.4 ± 0.7	2.4 ± 0.5
PD≥4mm (%)		25.2 ± 26.2	27.9 ± 28.4
Mean CAL (mm)		2.7 ± 0.9	2.6 ± 0.6
CAL≥4mm (%)		32.5 ± 26.0	36.0 ± 25.2
BOP (%)		23.1 ± 17.2	29.4 ± 21.4
PCR (%)		48.4 ± 19.6	54.6 ± 19.8

* N (%)

^†^Mean±SD

HbA1c, hemoglobin A1c; Cr, serum creatinine; TG, triglyceride; HDL-C, high-density lipoprotein cholesterol; LDL-C, low-density lipoprotein cholesterol; hs-CRP, high sensitive C-reactive protein; DTR-QOL, Diabetes Therapy-Related QOL; PPD, probing pocket depth; CAL, clinical attachment level; BOP, bleeding on probing; PCR, Plaque Control Record.

### Primary outcome

The target 3-month reduction of HbA1c levels of 0.8% or greater was not achieved. In the intention-to-treat analysis of the primary outcome, change in HbA1c levels at 3 months did not differ significantly between the periodontal treatment and control groups (Table 2). A per-protocol analysis evaluating the change in HbA1c levels also did not reveal between-group differences at 3 months (S1 Table).

**Table 2:** General conditions and periodontal parameters at follow-up in the primary analysis.

Parameter		3 months follow-up			
		Control group (N = 17)	Periodontal treatment group (N = 20)	Adjusted difference^†^ (95% CI)	P value^‡^
HbA1c (%)		7.7 ± 1.1*	7.4 ± 1.4	-0.16 (-0.56 to 0.23)	0.409
Glycated albumin (mg/dL)		19.9 ± 3.8	19.5 ± 4.4	-0.58 (-1.37 to 0.20)	0.140
Oxidative INDEX		0.2 ± 1.1	-0.9 ± 1.6	-1.19 (-2.03 to -0.35)	0.007
DTR-QOL	Factor 1	99.6 ± 52.0	109.0 ± 40.6	-0.99 (-31.37 to 29.39)	0.947
	Factor 2	34.5 ± 10.5	37.6 ± 9.6	0.23 (-4.49 to 4.94)	0.922
	Factor 3	20.0 ± 6.2	22.2 ± 6.2	-0.07 (-4.32 to 4.17)	0.972
	Factor 4	16.0 ± 6.1	17.9 ± 6.2	3.68 (0.25 to 7.10)	0.036
	Total	170.2 ± 65.7	186.6 ± 47.8	2.56 (-30.73 to 35.85)	0.877
Number of teeth present		24.5 ± 4.8	24.0 ± 6.3	0.08 (-0.36 to 0.51)	0.729
Mean PPD (mm)		2.5 ± 0.8	2.2 ± 0.6	-0.27 (-0.47 to -0.07)	0.011
PD≥4mm (%)		26.3 ± 29.6	20.5 ± 28.8	-6.75 (-17.88 to 4.39)	0.226
Mean CAL (mm)		2.7 ± 1.0	2.4 ± 0.6	-0.22 (-0.42 to -0.03)	0.024
CAL≥4mm (%)		34.5 ± 29.5	28.5 ± 27.0	-8.19 (-19.28 to 2.90)	0.142
BOP (%)		25.1 ± 16.7	24.1 ± 22.3	-8.72 (-19.38 to 1.93)	0.105
PCR (%)		42.6 ± 22.6	39.6 ± 17.6	-8.61 (-20.10 to 2.88)	0.137
Parameter		6 months follow-up			
		Control group (N = 17)	Periodontal treatment group (N = 20)	Adjusted difference (95% CI)	P value
HbA1c (%)		7.6 ± 1.1	7.4 ± 1.3	-0.07 (-0.48 to 0.34)	0.727
Glycated albumin (mg/dL)		18.0 ± 5.5	20.0 ± 4.2	-0.96 (-4.41 to 2.50)	0.577
Oxidative INDEX		0.2 ± 1.6	0.4 ± 1.6	-0.12 (-1.04 to 0.79)	0.786
DTR-QOL	Factor 1	60.9 ± 23.8	65.1 ± 23.9	13.43 (-4.13 to 31.01)	0.129
	Factor 2	36.3 ± 13.5	41.7 ± 13.4	4.64 (-1.81 to 11.09)	0.153
	Factor 3	18.6 ± 7.5	17.9 ± 11.4	1.06 (-3.39 to 5.51)	0.632
	Factor 4	17.1 ± 4.7	16.1 ± 8.2	3.24 (-0.67 to 7.16)	0.101
	Total	128.1 ± 47.8	135.2 ± 53.4	22.38 (-6.71 to 51.47)	0.127
Number of teeth present		24.5 ± 4.9	24.0 ± 6.3	0.12 (-0.35 to 0.59)	0.602
Mean PPD (mm)		2.6 ± 0.9	2.2 ± 0.5	-0.40 (-0.68 to -0.13)	0.006
PD≥4mm (%)		27.2 ± 30.1	19.4 ± 27.0	-9.57 (-21.03 to 1.87)	0.099
Mean CAL (mm)		2.8 ± 1.0	2.4 ± 0.6	-0.34 (-0.57 to -0.11)	0.005
CAL≥4mm (%)		36.9 ± 28.6	27.8 ± 27.0	-12.38 (-24.04 to -0.72)	0.038
BOP (%)		28.7 ± 22.0	23.1 ± 23.2	-13.10 (-24.24 to -1.96)	0.023
PCR (%)		44.9 ± 25.5	37.6 ± 16.4	-13.24 (-26.59 to 0.12)	0.052

* Mean±SD

^†^ Adjusted for insulin, medication and HbA1c

^‡^ Change in each parameter between the control and periodontitis group based on t-test from ANCOVA.

CI, Confidence interval; HbA1c, hemoglobin A1c; hs-CRP, high sensitive C-reactive protein; DTR-QOL, Diabetes Therapy-Related QOL; PPD, probing pocket depth; CAL, clinical attachment level; BOP, bleeding on probing; PCR, plaque control record.

### Secondary outcomes

A per-protocol analysis evaluating the change in HbA1c levels also did not reveal between-group differences at 6 months (S1 Table).

Change in glycated albumin levels also did not reveal between-group differences at 3 months in the intention-to-treat analysis (Table 2). Results at 6 months and in the per-protocol analysis were also similar (Table 2 and S1 Table).

In the intention-to-treat analysis of periodontal parameters, changes in mean PPD and mean CAL at 3 and 6 months significantly differed between the periodontal treatment and control groups (Table 2). Changes in the percentage of CAL≥4mm and BOP significantly differed between the periodontal treatment and control groups at 6 months (Table 2).

In the intention-to-treat analysis of oxidative stress, changes in oxidative-INDEX significantly differed between the periodontal treatment and control groups at 3 months (Table 2). Results in the per-protocol analysis were also similar (S1 Table).

At 3 months, the change in factor 4 (satisfaction with treatment of diabetes) in DTR-QOL significantly differed between the periodontal treatment and control groups (Table 2). The result in the per-protocol analysis was also similar (S1 Table).

We also compared the baseline characteristics between retained and dropout groups. There were significant differences in some parameters between the retained and dropout groups (S2 Table).

Of the 30 participants with medication data available at all study visits, 14 of 15 (93.3%) in the periodontal treatment group and 13 of 15 (86.7%) in the control group had no protocol-defined changes in diabetes medications during the study.

### Sub-group analysis

Change in HbA1c levels at 3 months in the sub-group [moderately poor glycemic control (HbA1c; 7.0 to 10.0%)] tended to show the difference between the periodontal treatment and control groups but did not reach the significant level (P = 0.070) (Table 3). Changes in mean PPD at 3 and 6 months significantly differed between the periodontal treatment and control groups (Table 3). The results of counterpart were shown in the S3 Table.

**Table 3:** General conditions and periodontal parameters at baseline and follow-up in the sub-group analysis [moderately poor glycemic control (HbA1c; 7.0 to 10.0%)].

Parameter		Baseline			
		Control group (N = 10)	Periodontal treatment group (N = 7)		
HbA1c (%)		8.1 ± 0.9*	7.8 ± 0.5		
Glycated albumin (mg/dL)		20.0 ± 3.1	20.0 ± 3.0		
Oxidative INDEX		0.4 ± 2.1	-0.2 ± 1.4		
DTR-QOL	Factor 1	55.7 ± 23.8	63.4 ± 16.9		
	Factor 2	30.8 ± 11.0	35.1 ± 12.7		
	Factor 3	19.6 ± 7.5	20.7 ± 8.7		
	Factor 4	17.3 ± 5.4	18.3 ± 7.7		
	Total	123.4 ± 39.8	137.6 ± 38.3		
Number of teeth present		26.3 ± 3.2	25.4 ± 2.6		
Mean PPD (mm)		2.4 ± 0.8	2.5 ± 0.5		
PD≥4mm (%)		22.5 ± 26.5	34.4 ± 34.0		
mean CAL (mm)		2.6 ± 1.0	2.8 ± 0.5		
CAL≥4mm (%)		31.4 ± 27.4	45.5 ± 27.3		
BOP (%)		19.3 ± 16.1	29.8 ± 23.1		
PCR (%)		46.0 ± 23.3	47.4 ± 19.2		
Parameter		3 months follow-up			
		Control group (N = 10)	Periodontal treatment group (N = 7)	Adjusted difference† (95% CI)	P value^‡^
HbA1c (%)		8.2 ± 1.1	7.4 ± 0.5	-0.41 (-0.86 to 0.04)	0.070
Glycated albumin (mg/dL)		20.8 ± 4.0	19.7 ± 2.8	-0.90 (-2.33 to 0.54)	0.201
Oxidative INDEX		0.4 ± 1.4	-0.8 ± 1.3	-0.50 (-1.99 to 0.99)	0.479
DTR-QOL	Factor 1	84.1 ± 39.2	102.7 ± 47.3	12.62 (-32.91 to 58.15)	0.560
	Factor 2	30.6 ± 7.7	35.9 ± 12.9	1.03 (-6.76 to 8.82)	0.780
	Factor 3	20.6 ± 4.8	20.0 ± 7.9	-1.30 (-7.68 to 5.08)	0.667
	Factor 4	12.9 ± 4.0	18.9 ± 8.0	4.93 (-0.20 to 10.07)	0.058
	Total	148.2 ± 46.9	177.4 ± 61.5	17.28 (-32.77 to 67.33)	0.469
Number of teeth present		25.9 ± 3.1	25.3 ± 2.7	0.34 (-0.43 to 1.11)	0.361
Mean PPD (mm)		2.5 ± 0.8	2.3 ± 0.6	-0.39 (-0.77 to -0.01)	0.046
PD≥4mm (%)		29.1 ± 31.1	23.9 ± 32.9	-16.30 (-38.98 to 6.37)	0.144
mean CAL (mm)		2.8 ± 1.0	2.6 ± 0.6	-0.37 (-0.73 to 0.00)	0.050
CAL≥4mm (%)		40.3 ± 31.2	36.0 ± 28.8	-18.53 (-40.47 to 3.41)	0.091
BOP (%)		27.3 ± 19.6	21.8 ± 18.7	-18.81 (-38.73 to 1.11)	0.062
PCR (%)		41.3 ± 24.3	37.4 ± 21.2	-7.19 (-20.98 to 6.59)	0.280
Parameter		6 months follow-up			
		Control group (N = 10)	Periodontal treatment group (N = 7)	Adjusted difference (95% CI)	P value
HbA1c (%)		8.1 ± 1.1	7.7 ± 0.5	0.04 (-0.60 to 0.69)	0.892
Glycated albumin (mg/dL)		17.6 ± 6.6	20.8 ± 3.5	-1.25 (-9.11 to 6.61)	0.637
Oxidative INDEX		0.7 ± 1.8	0.1 ± 1.3	-0.76 (-2.60 to 1.08)	0.386
DTR-QOL	Factor 1	55.1 ± 25.6	56.9 ± 27.7	16.66 (-15.76 to 49.08)	0.287
	Factor 2	36.4 ± 13.1	36.4 ± 12.3	2.33 (-8.84 to 13.50)	0.660
	Factor 3	18.8 ± 8.1	15.4 ± 13.4	0.43 (-7.00 to 7.86)	0.903
	Factor 4	15.6 ± 3.9	13.1 ± 11.8	0.63 (-5.70 to 6.95)	0.834
	Total	117.9 ± 52.6	118.1 ± 62.3	20.05 (-33.22 to 73.32)	0.431
Number of teeth present		25.9 ± 3.1	25.3 ± 2.7	0.34 (-0.43 to 1.11)	0.361
Mean PPD (mm)		2.5 ± 0.9	2.2 ± 0.7	-0.53 (-1.03 to -0.03)	0.040
PD≥4mm (%)		29.6 ± 32.6	24.3 ± 33.6	-17.43 (-41.54 to 6.67)	0.142
mean CAL (mm)		2.9 ± 1.0	2.6 ± 0.5	-0.48 (-0.96 to -0.02)	0.041
CAL≥4mm (%)		40.1± 31.0	39.0 ± 29.2	-16.00 (-40.18 to 8.19)	0.177
BOP (%)		28.0 ± 21.8	18.6 ± 18.1	-22.54 (-44.69 to -0.39)	0.047
PCR (%)		39.0 ± 27.1	32.4 ± 13.4	-10.33 (-26.90 to 6.24)	0.201

* Mean±SD

^†^ Adjusted for insulin, medication and HbA1c

^‡^ Change in each parameter between the control and periodontitis group based on t-test from ANCOVA.

CI, Confidence interval; HbA1c, hemoglobin A1c; DTR-QOL, Diabetes Therapy-Related QOL; PPD, probing pocket depth; CAL, clinical attachment level; BOP, bleeding on probing; PCR, plaque control record.

### Harms

The non-surgical periodontal treatment was a low-risk study, and no study-related serious adverse events occurred in all patients. No patients in the two groups required generalized periodontal rescue therapy during the study. In the control group, there were no patients who received the non-surgical periodontal treatment during the study.

## Discussion

To the best of our knowledge, this study is the first randomized clinical trial to investigate the effects of non-surgical periodontal treatment on systemic oxidative stress balance, QOL and glycemic control in patients with T2DM and chronic periodontitis. Despite its effectiveness in improving clinical measures of periodontitis, the periodontal therapy did not significantly change HbA1c levels at 3 or 6 months in the periodontal treatment group, and no differences in changes in HbA1c levels were observed between the two groups. Findings were similar in the intention-to-treat and the per-protocol analyses. Likewise, the periodontal treatment had no significant effect on glycated albumin. The results were similar to the previous large scale randomized clinical trial [35]. However, systemic oxidative stress balance and QOL score (satisfaction with diabetes treatment) were improved by the treatment and there were significant differences between the two groups at 3 months. Systemic oxidative stress affects the pathophysiology of diabetes [46]. On the other hand, satisfaction with diabetes treatment contributes to compliance [39]. Thus, periodontal treatment may be considered in patients with T2DM for its benefits of improving pathophysiology and compliance.

The primary outcome of glycemic control in this study was in contrast with three recently published meta analyses that showed a modest and significant reduction in HbA1c levels following periodontal treatment (-0.36% [95% CI, -0.54% to -0.19%], p < 0.0001 [12]; -0.48% (95% CI, -0.78% to 0.18%, p = 0.002 [47]; -0.40% [95% CI, -0.78% to -0.01%], P = 0.04 [8].) Some features of the present study may account for these differences. First, our trial enrolled participants who were under the care of two physicians for their diabetes and were within a range of HbA1c values that would be less likely to trigger a change in medications during the study period. We monitored changes in hypoglycemic medications during the study period like the previous study [35]. The number of patients with changes in diabetes medications during the study was quite small and was similar between the two groups. This aspect of the study design was critical, because medications may have a profound short-term influence on HbA1c levels, a point not adequately documented in previous studies. Second, we did not exclude patients with extremely lower or higher HbA1c levels before randomization. As a review suggests that the variety of HbA1c level in a study population may influence the results [48], we performed the sub-group analysis. It showed a modest (-0.41% [95% CI, -0.86% to 0.04%], P = 0.070) but not significant reduction in HbA1c levels following periodontal treatment. The values were within the range of previous studies [8, 12, 47]. However, the result needs to be interpreted with caution because the number of subgroup was small. Finally, the degree of improved periodontal parameters may be small in this study. The mean difference in change in mean PPD between the periodontal treatment and control groups was -0.27mm (95% CI, -0.47mm to -0.07mm, P = 0.011) at 3 months follow-up. In the meta-analysis, the mean difference was -0.39 mm (95% CI, -0.64 mm to -0.15 mm) after 3–4 months follow-up [8].

As a second outcome, we focused on systemic oxidative stress balance. Oxidative stress plays an important role in the pathophysiology of both periodontal disease and T2DM [15–25]. Oxidative stress appears to be important in the development and progression of diabetic complications [49]. For example, acute glucose variability induces endothelial dysfunction through oxidative stress, which can cause atherosclerosis [50]. On the other hand, periodontal treatment itself and adjunctive therapy for traditional periodontal treatment using antioxidant agents can improve systemic oxidative stress [24, 26, 51, 52]. The improvement of oxidative stress may contribute to improving glycemic control and reducing diabetes complications [53–56]. In this study, there were significant differences in the change in oxidative stress balance between the periodontal treatment and control groups at 3 months. However, the effects of periodontal treatment were transient. Long term effects on oxidative stress balance as well as glycemic control remain an issue [35].

Diabetes and its complications impair patients’ QOL [28, 30]. Improvement of these complications, e.g., periodontitis, may contribute to better QOL for diabetes patients. We reported that QOL scores (satisfaction with diabetes treatment) were improved following periodontal treatment. Treatment satisfaction influences compliance [39], which may contribute to the management of both diabetes and periodontitis. Thus, we have evaluated this new aspect in the present study, as recent reviews suggest that outcome measures in clinical trials should include QOL [9, 32]. However, the effect in this study was observed at 3 months, but not at 6 months. Further studies are needed to elucidate the factors involved in improving treatment compliance.

Patient characteristics were similar to other trials and were not population-specific. In this study, parameters such as age, HbA1c level, mean PPD, and mean CAL at baseline were within the range of the previous studies, which were similar with our design [35, 37, 57].

This study has some limitations. First, the sample size was determined by the previous study [37] but not the systemic reviews. Second, the non-surgical periodontal treatment was performed by general dentists, not periodontists. Since general dentists can play an important role in the community from the view point of public health, we did not choose periodontists for this study. These factors may underestimate the treatment effects. Third, not all of the clinical parameters were improved following periodontal treatment. Although PPD, CAL and BOP were significantly improved in the periodontal treatment group, significant improvement of PCR scores was not observed, indicating that changing self-care remains a challenge in T2DM patients.

Our trial, meanwhile, has a number of strengths. First, changes in diabetes medications were monitored during the follow-up visits. Second, periodontal treatment was conducted under supervision and resulted in a positive effect on a clinical measure of periodontitis among participants in the periodontal treatment group. Finally, the core laboratory responsible for the centralized analysis of blood samples analyzed the HbA1c values in a blinded manner.

## Conclusion

In T2DM patients, non-surgical periodontal treatment improved systemic oxidative stress balance and QOL, but did not decrease the levels of HbA1c as the main outcome at 3 months follow-up.

## Supporting information

S1 Table General conditions and periodontal parameters at baseline and follow-up in the per-protocol analysis. (DOCX)

S2 Table Differences between the retained and dropout groups at baseline. (DOCX)

S3 Table General conditions and periodontal parameters at baseline and follow-up in the sub-group analysis except for moderately poor glycemic control group. (DOCX)

S4 Table CONSORT checklist. (DOC)

S1 Protocol Protocol (in Japanese). (DOC)

S2 Protocol Protocol (in English). (DOC)
