# Supplementary material for: The effects of non-surgical periodontal treatment on glycemic control, oxidative stress balance and quality of life in patients with type 2 diabetes: A randomized clinical trial
Source: PLoS One. 2017 Nov 16;12(11):e0188171. doi: 10.1371/journal.pone.0188171 (PMC5689834; doi:10.1371/journal.pone.0188171)
Supplement: S1 Table — (DOCX) [file pone.0188171.s001.docx]

**S1 Table. General conditions and periodontal parameters at baseline and follow-up in the per-protocol analysis.**

|  |  | Baseline | |  |  |
| --- | --- | --- | --- | --- | --- |
| Parameter |  | Control group (N=17) | Periodontal treatment group (N=20) |  |  |
| HbA1c (%) |  | 7.7 ± 1.2* | 7.5 ± 1.7 |  |  |
| Glycated albumin (mg/dL) |  | 19.3 ± 3.4 | 19.3 ± 4.7 |  |  |
| Oxidative INDEX |  | 0.0 ± 1.7 | 0.1 ± 1.5 |  |  |
| DTR-QOL | Factor 1 | 61.4 ± 22.2 | 72.6 ± 26.0 |  |  |
|  | Factor 2 | 33.7 ± 11.3 | 36.6 ± 10.6 |  |  |
|  | Factor 3 | 19.2 ± 7.3 | 21.5 ± 6.7 |  |  |
|  | Factor 4 | 19.5±5.0 | 17.8±5.6 |  |  |
|  | Total | 133.8±39.0 | 148.7±39.0 |  |  |
| Number of teeth present |  | 24.8 ± 4.8 | 24.3 ± 6.2 |  |  |
| Mean PPD (mm) |  | 2.4 ± 0.7 | 2.4 ± 0.5 |  |  |
| PD≥4mm (%) |  | 25.2 ± 26.2 | 27.9 ± 28.4 |  |  |
| mean CAL (mm) |  | 2.7 ± 0.9 | 2.6 ± 0.6 |  |  |
| CAL≥4mm (%) |  | 32.5 ± 26.0 | 36.0 ± 25.2 |  |  |
| BOP (%) |  | 23.1 ± 17.2 | 29.4 ± 21.4 |  |  |
| PCR (%) |  | 48.4 ± 19.6 | 54.6 ± 19.8 |  |  |
|  |  | 3 months follow-up | |  |  |
| Parameter |  | Control group (N=14) | Periodontal treatment group (N=17) | Adjusted difference^†^  (95% CI) | P value^‡^ |
| HbA1c (%) |  | 7.6 ± 1.1 | 7.1 ± 1.2 | 0.08  (-0.37 to 0.53) | 0.715 |
| Glycated albumin (mg/dL) |  | 20.6 ± 3.9 | 18.8 ± 3.6 | -0.66  (-1.63 to 0.32) | 0.178 |
| Oxidative INDEX |  | 0.0 ± 0.8 | -1.4 ± 0.7 | -1.52  (-2.40 to -0.63) | 0.002 |
| DTR-QOL | Factor 1 | 94.4 ± 42.3 | 109.3 ± 38.9 | 4.69  (-27.49 to 36.86) | 0.767 |
|  | Factor 2 | 33.8 ± 9.9 | 40.2 ± 7.3 | 2.62  (-2.23 to 7.47) | 0.278 |
|  | Factor 3 | 20.1 ± 6.1 | 22.4 ± 6.5 | 0.48  (-3.89 to 4.84) | 0.825 |
|  | Factor 4 | 15.9±5.9 | 18.6±5.8 | -4.30  (-8.32 to -0.28) | 0.037 |
|  | Total | 164.4±56.4 | 192.4±44.8 | 3.48  (-29.77 to 36.73) | 0.831 |
| Number of teeth present |  | 25.5 ± 3.4 | 25.0 ± 4.5 | 0.13  (-0.44 to 0.70) | 0.643 |
| Mean PPD (mm) |  | 2.3 ± 0.7 | 2.1 ± 0.5 | -0.26  (-0.45 to -0.14) | 0.039 |
| PD≥4mm (%) |  | 23.5 ± 28.3 | 16.7 ± 25.2 | -8.99  (-23.05 to 5.07) | 0.200 |
| mean CAL (mm) |  | 2.6 ± 0.9 | 2.3 ± 0.5 | -0.25  (-0.50 to -0.01) | 0.044 |
| CAL≥4mm (%) |  | 32.2 ± 29.0 | 25.6 ± 24.4 | -9.72  (-23.55 to 4.12) | 0.161 |
| BOP (%) |  | 25.1 ± 17.1 | 22.4 ± 22.4 | -9.96  (-22.27 to 2.36) | 0.109 |
| PCR (%) |  | 43.4 ± 23.1 | 40.2 ± 18.7 | 0.50  (-12.07 to 13.07) | 0.936 |
|  |  | 6 months follow-up | |  |  |
| Parameter |  | Control group (N=13) | Periodontal treatment group (N=15) | Adjusted difference (95% CI) | P value |
| HbA1c (%) |  | 7.5 ± 0.9 | 7.1 ± 0.8 | 0.31  (-0.16 to 0.77) | 0.188 |
| Glycated albumin (mg/dL) |  | 19.6 ± 3.6 | 19.5 ± 3.0 | 0.87  (-0.20 to 1.93) | 0.106 |
| Oxidative INDEX |  | 0.1 ± 1.5 | 0.3 ± 1.3 | -0.12  (-1.14 to 0.89) | 0.804 |
| DTR-QOL | Factor 1 | 64.9 ± 19.6 | 74.0 ± 10.4 | 2.60  (-4.57 to 9.77) | 0.461 |
|  | Factor 2 | 34.3 ± 12.7 | 41.5 ± 8.0 | 1.94  (-4.32 to 8.19) | 0.528 |
|  | Factor 3 | 19.3 ± 6.4 | 19.9 ± 7.9 | 0.38  (-2.92 to 3.68) | 0.814 |
|  | Factor 4 | 18.1±4.9 | 18.7±6.2 | -1.74  (-4.04 to 0.55) | 0.130 |
|  | Total | 136.5±35.5 | 154.0±26.0 | 3.17  (-8.60 to 14.94) | 0.582 |
| Number of teeth present |  | 25.7 ± 3.6 | 24.6 ± 4.7 | 0.14  (-0.56 to 0.84) | 0.685 |
| Mean PPD (mm) |  | 2.3 ± 0.5 | 2.1 ± 0.5 | -0.35  (-0.70 to -0.01) | 0.045 |
| PD≥4mm (%) |  | 19.6 ± 22.8 | 16.3 ± 23.7 | -11.71  (-27.67 to 4.25) | 0.143 |
| mean CAL (mm) |  | 2.5 ± 0.4 | 2.4 ± 0.5 | -0.32  (-0.64 to 0.01) | 0.054 |
| CAL≥4mm (%) |  | 30.8 ± 22.2 | 26.4 ± 25.4 | -14.42  (-30.23 to 1.40) | 0.072 |
| BOP (%) |  | 26.1 ± 20.4 | 20.8 ± 25.0 | -13.85  (-28.66 to -0.96) | 0.065 |
| PCR (%) |  | 47.2 ± 25.2 | 35.8±17.6 | -6.49  (-21.72 to 8.75) | 0.387 |

* Mean±SD

† Adjusted for insulin, medication and HbA1c

‡ Change in each parameter between the control and periodontitis group based on t-test from ANCOVA.

CI, Confidence interval; HbA1c, hemoglobin A1c; hs-CRP, high sensitive C-reactive protein; DTR-QOL, Diabetes Therapy-Related QOL; PPD, probing pocket depth; CAL, clinical attachment level; BOP, bleeding on probing; PCR, plaque control record.
