# Supplementary material for: The effects of non-surgical periodontal treatment on glycemic control, oxidative stress balance and quality of life in patients with type 2 diabetes: A randomized clinical trial
Source: PLoS One. 2017 Nov 16;12(11):e0188171. doi: 10.1371/journal.pone.0188171 (PMC5689834; doi:10.1371/journal.pone.0188171)
Supplement: S2 Table — (DOCX) [file pone.0188171.s002.docx]

**S2 Table. Differences between the retained and dropout groups at baseline.**

| Parameter |  | Retained  group (N=28) | Dropout  group (N=9) | P value |
| --- | --- | --- | --- | --- |
| Sex | Male | 23 (82.1)* | 5 (55.6) | 0.123^†^ |
| Age (years) |  | 62.8±10.2^‡^ | 59.1±11.4 | 0.364^§^ |
| Medical history except for diabetes (number) | >2 | 27 (96.4) | 8 (88.9) | 0.432 |
| Medication except for diabetes (number) | >2 | 27 (96.4) | 9 (100.0) | 0.757 |
| BMI (kg/m^2^) |  | 25.1±3.4 | 29.1±4.6 | 0.009 |
| Exercise time (hour/week) |  | 2.3±2.7 | 0.2±0.7 | 0.001 |
| Smoker (person) |  | 6 (21.4) | 2 (22.2) | 0.643 |
| Drinker (person) |  | 14 (50.0) | 4 (44.4) | 0.538 |
| Frequency of toothbrushing (times/day) | ≥ two times | 14 (50.0) | 5 (55.6) | 0.538 |
| Use of interdental brush (number) |  | 11 (39.2) | 3 (33.4) | 0.536 |
| HbA1c (%) |  | 7.2±1.0 | 8.7±2.2 | 0.070 |
| Glycated albumin (mg/dL) |  | 19.0±3.2 | 20.5±6.2 | 0.500 |
| Cr (mg/dL) |  | 1.0±0.4 | 0.7±0.2 | 0.123 |
| TG (mg/dL) |  | 142.0±71.4 | 249.8±136.0 | 0.047 |
| HDL-C (mg/dL) |  | 54.3±11.3 | 49.0±15.9 | 0.280 |
| LDL-C (mg/dL) |  | 95.9±21.3 | 99.0±31.4 | 0.740 |
| Hs-CRP(ng/mL) |  | 4789.9±1148.5 | 4647.4±2473.5 | 0.871 |
| Oxidative INDEX |  | -0.4±1.2 | 1.4±1.9 | 0.002 |
| DTR-QOL | Factor 1 | 71.6±26.1 | 55.1±18.6 | 0.090 |
|  | Factor 2 | 36.8±10.2 | 30.3±11.9 | 0.119 |
|  | Factor 3 | 20.8±7.1 | 19.6±6.7 | 0.659 |
|  | Factor 4 | 19.5±5.1 | 15.6±5.1 | 0.051 |
|  | Total | 148.6±38.3 | 120.6±36.1 | 0.060 |
| Number of teeth present |  | 25.5±3.9 | 21.4±8.5 | 0.208 |
| Mean PPD (mm) |  | 2.2±0.4 | 2.9±0.8 | 0.025 |
| PD≥4mm (%) |  | 20.9±22.2 | 44.5±34.1 | 0.080 |
| Mean CAL (mm) |  | 2.4±0.5 | 3.3±1.1 | 0.044 |
| CAL≥4mm (%) |  | 29.5±21.2 | 49.5±31.8 | 0.107 |
| BOP (%) |  | 22.4±18.5 | 39.5±18.1 | 0.021 |
| PCR (%) |  | 49.9±16.1 | 57.5±28.7 | 0.317 |

* N (%)

† χ2 test

‡ Mean±SD

§ t-test

HbA1c, hemoglobin A1c; Cr, serum creatinine; TG, Triglyceride; HDL-C, high-density lipoprotein cholesterol; LDL-C, low-density lipoprotein cholesterol; hs-CRP, high sensitive C-reactive protein; Daibetes Therapy-Related QOL, DTR-QOL; PPD, probing pocket depth; CAL, clinical attachment level; BOP, bleeding on probing; PCR, Plaque Control Record.
