# Supplementary material for: The effects of non-surgical periodontal treatment on glycemic control, oxidative stress balance and quality of life in patients with type 2 diabetes: A randomized clinical trial
Source: PLoS One. 2017 Nov 16;12(11):e0188171. doi: 10.1371/journal.pone.0188171 (PMC5689834; doi:10.1371/journal.pone.0188171)
Supplement: S3 Table — (DOCX) [file pone.0188171.s003.docx]

**S3 Table. General conditions and periodontal parameters at baseline and follow-up in the sub-group analysis except for moderately poor glycemic control group.**

|  |  | Baseline | |  |  |
| --- | --- | --- | --- | --- | --- |
| Parameter |  | Control group  (N=10) | Periodontal  treatment group  (N=7) |  |  |
| HbA1c (%) |  | 7.1±1.4* | 7.3±2.1 |  |  |
| Glycated albumin (mg/dL) |  | 18.3±3.7 | 18.9±5.5 |  |  |
| Oxidative INDEX |  | -0.6±0.7 | 0.3±1.6 |  |  |
| DTR-QOL | Factor 1 | 69.4±18.4 | 77.9±30.7 |  |  |
|  | Factor 2 | 37.9±11.0 | 37.3±9.7 |  |  |
|  | Factor 3 | 18.7±7.4 | 21.9±5.6 |  |  |
|  | Factor 4 | 22.6±2.0 | 17.5±4.4 |  |  |
|  | Total | 148.6±35.4 | 154.6±39.6 |  |  |
| Number of teeth present |  | 22.7±6.2 | 23.6±7.5 |  |  |
| Mean PPD (mm) |  | 2.4±0.6 | 2.3±0.5 |  |  |
| PD≥4mm (%) |  | 29.1±27.4 | 24.4±25.8 |  |  |
| mean CAL (mm) |  | 2.7±0.9 | 2.4±0.6 |  |  |
| CAL≥4mm (%) |  | 34.2±25.8 | 30.8±23.5 |  |  |
| BOP (%) |  | 28.6±18.5 | 29.2±21.4 |  |  |
| PCR (%) |  | 51.8±13.7 | 58.5±19.8 |  |  |
|  |  | 3 months follow-up | |  |  |
| Parameter |  | Control group  (N=10) | Periodontal  treatment group  (N=7) | Adjusted difference^†^ (95% CI) | P value^‡^ |
| HbA1c (%) |  | 7.0±0.7 | 7.3±1.8 | 0.23  (-0.80 to 1.26) | 0.599 |
| Glycated albumin (mg/dL) |  | 18.8±3.3 | 19.4±5.1 | -0.27  (-1.93 to 1.40) | 0.710 |
| Oxidative INDEX |  | 0.0±0.5 | -0.9±1.8 | -1.06  (-2.30 to 0.19) | 0.083 |
| DTR-QOL | Factor 1 | 121.9±62.7 | 112.3±38.2 | 11.50  (-88.78 to 111.78) | 0.788 |
|  | Factor 2 | 40.1±12.0 | 38.5±7.7 | 2.83  (-9.51 to 15.17) | 0.595 |
|  | Factor 3 | 19.1±8.2 | 23.3±5.1 | 7.50  (-7.10 to 22.10) | 0.256 |
|  | Factor 4 | 20.4±6.0 | 17.4±5.4 | -0.33  (-13.52 to 12.85) | 0.953 |
|  | Total | 201.6±79.2 | 191.5±40.6 | 21.50  (-85.98 to 128.98) | 0.642 |
| Number of teeth present |  | 22.6±6.3 | 23.3±7.6 | -0.17  (-0.91 to 0.58) | 0.604 |
| Mean PPD (mm) |  | 2.4±0.8 | 2.1±0.5 | -0.10  (-0.67 to 0.46) | 0.668 |
| PD≥4mm (%) |  | 22.4±29.2 | 18.7±27.6 | 4.40  (-18.49 to 27.29) | 0.655 |
| mean CAL (mm) |  | 2.6±1.0 | 2.2±0.6 | -0.04  (-0.60 to 0.53) | 0.881 |
| CAL≥4mm (%) |  | 26.2±26.8 | 24.4±26.3 | 1.92  (-22.88 to 26.72) | 0.856 |
| BOP (%) |  | 21.9±12.1 | 25.3±24.6 | -8.47  (-30.90 to 13.97) | 0.391 |
| PCR (%) |  | 44.5±21.7 | 40.8±16.1 | -10.83  (-45.89 to 24.23) | 0.478 |
|  |  | 6 months follow-up | |  |  |
| Parameter |  | Control group (N=7) | Periodontal treatment group (N=13) | Adjusted difference (95% CI) | P value |
| HbA1c (%) |  | 7.1±0.8 | 7.3±1.5 | 0.05  (-1.20 to 1.30) | 0.922 |
| Glycated albumin (mg/dL) |  | 18.7±3.9 | 19.5±4.5 | 1.61  (-2.14 to 5.36) | 0.319 |
| Oxidative INDEX |  | -0.4±1.2 | 0.6±1.7 | -0.62  (-1.78 to 0.54) | 0.226 |
| DTR-QOL | Factor 1 | 69.1±19.6 | 69.5±21.5 | -4.30  (-24.64 to 16.04) | 0.610 |
|  | Factor 2 | 36.1±15.2 | 44.5±13.6 | -1.00  (-14.67 to 12.67) | 0.858 |
|  | Factor 3 | 18.3±7.3 | 19.2±10.6 | 3.10  (-8.43 to 14.63) | 0.520 |
|  | Factor 4 | 19.1±5.3 | 17.6±5.3 | 1.70  (-2.94 to 6.34) | 0.390 |
|  | Total | 142.7±38.9 | 144.3±48.1 | -0.50  (-35.50 to 34.50) | 0.972 |
| Number of teeth present |  | 22.4±6.4 | 23.3±7.6 | -0.20  (-1.06 to 0.66) | 0.576 |
| Mean PPD (mm) |  | 2.6±1.0 | 2.2±0.5 | -0.03  (-0.50 to 0.44) | 0.882 |
| PD≥4mm (%) |  | 23.7±28.1 | 16.7±23.9 | 0.64  (-27.04 to 28.31) | 0.955 |
| mean CAL (mm) |  | 2.7±1.1 | 2.3±0.6 | 0.07  (-0.36 to 0.50) | 0.700 |
| CAL≥4mm (%) |  | 32.3±26.4 | 21.7±24.9 | -1.73  (-34.63 to 31.18) | 0.898 |
| BOP (%) |  | 29.6±24.0 | 25.5±25.8 | -4.80  (-36.24 to 26.64) | 0.711 |
| PCR (%) |  | 53.3±22.2 | 40.4±17.6 | -23.96  (-62.26 to 14.34) | 0.169 |

* Mean±SD

† Adjusted for insulin, medication and HbA1c

‡ Change in each parameter between the control and periodontitis group based on t-test from ANCOVA.

CI, Confidence interval; HbA1c, hemoglobin A1c; DTR-QOL, Diabetes Therapy-Related QOL; PPD, probing pocket depth; CAL, clinical attachment level; BOP, bleeding on probing; PCR, plaque control record.
