# Supplementary material for: The effects of non-surgical periodontal treatment on glycemic control, oxidative stress balance and quality of life in patients with type 2 diabetes: A randomized clinical trial
Source: PLoS One. 2017 Nov 16;12(11):e0188171. doi: 10.1371/journal.pone.0188171 (PMC5689834; doi:10.1371/journal.pone.0188171)
Supplement: S1 Protocol — (DOC) [file pone.0188171.s005.doc]

**S1 Protocol. Protocol (in Japanese).**

＜臨床研究実施計画書＞

臨床研究課題名：2型糖尿病患者に対する歯科保健指導および歯周治療の効果の検証

研究責任者：森田　学

岡山大学大学院医歯薬学総合研究科社会環境生命科学専攻総合社会医科学講座

予防歯科学分野

〒700－8558　岡山市北区鹿田町2－5－1

電話番号（内線）086－235－6710（6710）

FAX番号　086－235－6714

E-mail　mmorita@md.okayama-u.ac.jp

緊急連絡先：086－235－6712（医局番号）

研究実施予定期間：平成26年3月1日～平成28年3月31日

「2型糖尿病患者に対する歯科保健指導および歯周治療の効果の検証」

研究実施計画書

１．研究の背景

平成24年8月に成立した「歯科口腔保健の推進に関する法律」に基づく「歯科口腔保健の推進に関する基本的事項」には、口腔の状態と全身の健康との関係、歯科疾患と生活習慣との関係、歯科口腔保健と医療費との関係及び歯科に係るより効果的な予防・治療法等についての研究を推進することと記載されている。特に、歯周病と糖尿病との密接な関係が明らかになりつつあることから、糖尿病患者における歯科・医科連携モデル事業のイノベーションが求められている。

２．研究の目的と必要性

本研究では、岡山大学病院に来院した2型糖尿病患者を対象に、歯科保健指導群（プラークコントロール指導のみの群）と歯周治療群（プラークコントロール指導に加えて歯周治療を実施する群）の2群に分けて、歯周治療（全顎的な歯石除去）が終わってから6ヶ月間の検査（臨床検査、血液生化学検査）結果やQOLの変化に及ぼす影響を比較検討することを目的とする。

糖尿病患者における医科・歯科連携の現状は、協力医療機関と協力歯科医療機関とがお互いに紹介し合う程度であり、その臨床的意義は不明である。本研究の成果は、その連携内容に臨床的意義（糖尿病の病態が歯周治療によって改善することの裏付け）を持たせることにある。このことは、将来の厚生労働行政の施策において「糖尿病等の患者に対する歯科支援加算（仮）」等、保険診療への導入の根拠としての活用が期待される。

３．医薬品・医療機器の概要

該当なし。

４．対象患者

　　（１）選択基準：

以下の基準を全て満たす患者を対象とする。

1. 2型糖尿病の診断を受けた者
2. 6ヶ月間通院が可能な者
3. 健康保険の加入者
4. 同意取得時において年齢が30歳以上の患者
5. 本研究の参加にあたり十分な説明を受けた後、十分な理解の上、患者本人の自由意思による文書同意が得られた患者
6. 外来患者

（２）除外基準：

以下のいずれかに抵触する患者は本試験に組み入れないこととする。

- - - - 1. 歯科治療中の患者
        2. 妊娠中あるいは妊娠の可能性がある女性
        3. その他、研究責任者、研究分担者が被験者として不適当と判断した患者

５．被験者に説明し同意を得る方法

臨床研究審査委員会で承認の得られた同意説明文書を患者に渡し、文書および口頭による十分な説明を行い、患者の自由意思による同意を文書で得る。また、患者の同意に影響を及ぼすような実施計画等の変更が行われるときは、速やかに患者に情報提供し、研究に参加するか否かについて患者の意思を改めて確認するとともに、事前に臨床研究審査委員会の承認を得て同意説明文書等の改訂を行い、患者の再同意を得る。

６．研究の方法

以下の項目について記載すること。

（１）研究の種類・デザイン

　ランダム化比較研究

（２）研究のアウトライン

| **評価内容**  ・HbA1cの比較  ・歯周状態、血糖、酸化ストレス、QOLの比較  **＜歯科保健指導群＞**  歯磨き指導  研究参加者の募集  **＜歯周治療群＞**  歯科保健指導群の内容  ＋歯石の除去・術者みがき・機械的歯面清掃 |
| --- |

図．研究のフローチャート

岡山大学病院の腎・免疫・内分泌代謝内科において研究の参加に同意の得られた2型糖尿病患者を予防歯科の外来に紹介し、研究グループの一人が乱数表で歯科保健指導群と歯周治療群の2群に分ける（患者には、割り付けの結果を診査者に知らせないように依頼する）。その後、割り付けの結果を知らない2人の歯科医師が歯周検査を実施する。そして、さらに別の歯科医師が歯科保健指導および歯周治療を担当する。なお、歯周検査については、あらかじめ、術者間、術者内の一致度において、カッパ値（信頼性評価指標の一つ）が0.8を超えるまでキャリブレーションを繰り返す。

歯周検査では、歯周ポケットの深さ、クリニカルアタッチメントレベル（歯冠と歯根の境から歯周ポケットの底部までの距離）、プロービング時出血（歯周ポケットの深さを測定したときに認められる出血）の有無、および歯垢付着の程度を調べる。

一方、腎・免疫・内分泌代謝内科では、通常の診療の一環として、血液検査を行う（腎・免疫・内分泌代謝内科と予防歯科の受診は同日になるように調整する）。血液検査では、グリコヘモグロビン（HbA1c）、およびグリコアルブミンを評価する。また、診療時に採取された血液のうち、残余(医療廃棄物として処分されるもの）を研究グループが受け取り、酸化ストレスバランスを定量する。

上記した診査の一部は研究開始時、3ヶ月後、および6ヶ月後の計3回行い、歯周治療は研究開始時から2ヶ月経過するまでの間に完了させる。また、歯科保健指導は、両群ともに研究開始時、3ヶ月後、および6ヶ月後に行う。なお、期間中に糖尿病治療に変更があった場合は、その内容を記録する。

（３）被験者の研究参加予定期間

　　　6ヶ月間

（４）試験薬の用法・用量、投与期間

　　　該当なし。

（５）試験薬の剤形・含有量、性状、包装、表示、貯法

　　　該当なし。

（６）併用薬（療法）に関する規定

該当なし。

（７）休薬の方法

　　　 該当なし。

（８）試験薬の管理・交付手順

　　　 該当なし。

（９）服薬指導情報

　　　 該当なし。

（10）症例登録、割付方法

被験者の登録方法：

研究責任者あるいは研究分担者は、１）文書による同意を取得する。２）研究責任者が保管する被験者識別コードリストに、同意取得日および被験者と被験者識別コードを対応させるために必要な事項を記載する。３）被験者識別コードを用いた症例登録書を、研究事務局（予防歯科学分野医局）に提出する。４）同意撤回、中止、脱落等が生じた時は、速やかに報告する。

被験者の割付方法：

被験者の各治療群への割付は、乱数表で行う。層化ブロックランダム割り付け法を用いて、ベースラインの血清HbA1c（8％以上と未満）・インスリン療法の有無・薬剤の種類（2種類以下と3種類以上）で調整する。

（11）研究終了後の対応

本研究終了後は、この研究で得られた成果も含めて、研究責任者は被験者に対し最も適切と考える医療を提供する。

７．評価項目

　　　（１）主要評価項目

　　　　　　血清HbA1c（NGSP値）

　　　（２）副次的評価項目

　　　　　　歯周状態（現在歯数、歯周ポケットの深さ、クリニカルアタッチメントレベル、

プロービング時出血の有無、歯垢付着指数）

　　　　　　血液学的指標（グリコアルブミン）

　　　　　　血液生化学的指標（酸化ストレスバランス）

QOL

　　　（３）安全性評価項目

　　　　　　該当なし。

８．観察および検査項目

（１）患者背景：カルテ番号、患者イニシャル、性別、生年月日、人種、入院・外来の別、

身長、体重、合併症、既往歴、現病歴、前治療など

（２）自他覚症状の確認：問診等により確認する。症状日誌がある場合は参考にする。

（３）血液検査：通常の診療時と同じ項目を測定する。

（４）血液生化学検査：通常の診療時と同じ項目を測定する。さらに、酸化ストレスに関わる因子についても評価を行い、口腔と全身との関連性を確認する。

（５）歯周検査：現在歯数、歯周ポケットの深さ、クリニカルアタッチメントレベル、プロービング時出血の有無および歯垢付着指数を評価する。

（６）QOLに関するアンケート調査

　　　　　　　　　表．スケジュール

| 項　目 | | 開始日 | 歯周治療期間 | 観察期間 | |  |
| --- | --- | --- | --- | --- | --- | --- |
| 時　期 | | 0週 | 2ヵ月以内 | 3ヵ月後 | 6ヵ月後 | |
| 受　診 | | 受診1 | 受診2－5 | 受診6 | 受診7 | |
| 同意取得 | | ○ |  |  |  | |
| 患者背景の確認^a^ | | ○ |  |  |  | |
| 歯科保健指導 | |  |  |  |  | |
| 歯周治療 | |  |  |  |  | |
| 自他覚症状の確認 | | ○ |  | ● | ● | |
| QOLに関する  アンケート調査 | | ○ |  | ● | ● | |
| 臨  床  検  査 | 血液学的検査^ｂ^ | ○ |  | ● | ● | |
|  | 血液生化学検査^ｃ^ | ○ |  | ● | ● | |
| 歯周検査 | | ○ |  | ● | ● | |

○印は歯科介入前に行う項目、●印は歯科介入後に行う項目

a：患者背景とは、年齢・性別・既往症・合併症など患者さんの医療における特徴のことです。

b: 血液学的検査として、通常の診療時と同じ項目を測定します。

c: 血液生化学検査として、通常の診療時と同じ項目を測定します。さらに、酸化ストレスに関わる因子についても評価を行います。これらは口腔と全身との関連性を確認するために行います。

９．中止基準

- 1. 被験者から研究参加の辞退の申し出や同意の撤回があった場合
  2. 登録後に適格性を満足しないことが判明した場合
  3. 合併症の増悪により研究の継続が困難な場合
  4. 妊娠が判明した場合
  5. 研究全体が中止された場合
  6. 割り付け後に歯科保健指導群で歯科治療が必要になった場合
  7. その他の理由により、医師が研究を中止することが適当と判断した場合

１０．有害事象発生時の取扱

（１）有害事象発生時の被験者への対応

被験者に有害事象が生じ、治療が必要であると認めるときは、その旨を被験者に通知し、有害事象に対して適切な医療を提供する。

（２）重篤な有害事象の報告

　　　重篤な有害事象の発生を認めたとき又はその発生のおそれがあると認めたときは、直ちに岡山大学病院長に報告する。

１１．実施計画書からの逸脱の報告

GCPに準じ、以下の事項について記載する。

- 研究責任者または研究分担者は、臨床研究審査委員会の事前の審査に基づく病院長の承認を得る前に、研究実施計画書からの逸脱あるいは変更を行ってはならない。
- 研究責任者または研究分担者は、緊急回避等のやむを得ない理由により、臨床研究審査委員会の事前の承認を得る前に、研究実施計画書からの逸脱あるいは変更を行うことができる。その際には、研究責任者または研究分担者は、逸脱または変更の内容および理由ならびに研究実施計画書等の改訂が必要であればその案を速やかに、臨床研究審査委員会に提出し、臨床研究審査委員会および病院長の承認を得るものとする。
- 研究責任者または研究分担者は、研究実施計画書からの逸脱があった場合は、逸脱事項をその理由とともに全て記録する。

１２．研究の終了、中止、中断

（１）研究の終了

研究の終了時には、研究責任者は、速やかに研究終了報告書を病院長に提出する。

（２）研究の中止、中断

研究責任者は、以下の事項に該当する場合は研究実施継続の可否を検討する。

１）被験者のリクルートが困難で予定症例を達成することが到底困難であると判断されたとき。

２）臨床研究審査委員会により、実施計画等の変更の指示があり、これを受入れることが困難と判断されたとき。

なお、研究の中止または中断を決定した時は、速やかに病院長にその理由とともに

文書で報告する。

１３．研究実施期間

平成２６年３月１日から平成２８年３月３１日

１４．データの集計および統計解析方法

データの解析を以下の観点から行う。

１） 糖尿病患者におけるプラークコントロール指導の役割：歯科保健指導群における研究開始時と再評価時との比較から、プラークコントロール指導の意義をQOLへの影響も含めて検討する。

２）歯周治療の意義：歯科保健指導群と歯周治療群との比較から、糖尿病患者に対する歯周治療の効果を見定める。

１５．目標症例数および設定根拠

目標症例数20例（2群で40例）

糖尿病患者に対して歯周治療を行った論文（参考文献参照）の血清HbA1c値から、20人の症例数を算出した。*t*検定を使用する場合のサンプルサイズを決定するため、SPSS

Samplepower（IBM、Tokyo）を用いて計算した。この場合、αは0.05、βは0.20、および両側検定として、歯科保健指導群のHｂA1ｃ予測値を7.31％、歯周治療群のHbA1c予測値を6.51％、想定する分散標準偏差は0.8として、歯周治療によって血清HbA1cの平均が0.8％以上異なるときに有意であると仮定した。その結果、17人となり、さらに15％の脱落を考慮して20人とした。

１６．被験者の人権および安全性・不利益に対する配慮

（１）人権への配慮（プライバシーの保護）

　対象者のデータをコード化し、岡山大学の分析者に本人が識別できないようにして結果を渡す。なお、診査表と質問表,各種検査のデータの結合を行うために同一の受診者には同じ記号を付与する。

また、研究終了後、これらの資料は破棄する。

（２）安全性・不利益への配慮

　被験者への健康被害の補償のために、保険その他の必要な措置を講じていない。

理由：採血時に若干の痛みを伴う可能性があるのみで、健康被害が出る想定をしていないため。

１７．患者の費用負担

　通常の診療の範囲内である。

１８．臨床研究に参加する事により期待される利益，起こり得る危険

この研究に参加することによる利益として、謝礼として1回の診療あたり2,000円のクオカードが渡される。ただし、不利益として、各種検査に時間がかかる。また、危険性はない。

１９．健康被害の補償

　被験者への健康被害の補償のために、保険その他の必要な措置を講じていない。

理由：本研究は通常の診療の範囲内で行われるため、健康被害が出る想定をしていない。

２０．GCP及びヘルシンキ宣言への対応

　本研究はGCPを準用するものとする。また、ヘルシンキ宣言（2008年改訂）を遵守して実施する。

２１．記録の保存

研究責任者は、研究等の実施に係わる必須文書（申請書類の控え、病院長からの通知文書、各種申請書・報告書の控、被験者識別コードリスト、同意書、症例報告書等の控、その他データの信頼性を保証するのに必要な書類または記録など）を保存し、研究発表後に廃棄する。

２２．研究結果の公表

　研究結果は、平成28年4月30日までに報告書等にまとめて病院長に報告する。また、論文発表や学会発表も行う。なお、研究結果を公開する際には、被験者を特定できる個人情報等は開示しない。

２３．研究組織

所属：岡山大学大学院医歯薬学総合研究科社会環境生命科学専攻総合社会医科学講座

予防歯科学分野　　　　　　　　職名：教授　　氏名：○　森田　学

所属：岡山大学大学院医歯薬学総合研究科社会環境生命科学専攻総合社会医科学講座

公衆衛生学分野　　　　　　　　職名：教授　　氏名：荻野景規

所属：岡山大学大学院医歯薬学総合研究科病態制御科学専攻病態機構学講座

腎・免疫・内分泌代謝内科学分野　　　　職名：准教授　氏名：和田　淳

所属：岡山大学病院予防歯科　　　　　　　　　職名：講師　　氏名：友藤孝明

所属：岡山大学病院予防歯科　　　　　　　　　職名：講師　　氏名：江國大輔

所属：岡山大学大学院医歯薬学総合研究科社会環境生命科学専攻総合社会医科学講座

予防歯科学分野　　　　　　　　　　　　職名：助教　　氏名：東　哲司

所属：岡山大学病院新医療研究開発センター　　職名：助教　　氏名：丸山貴之

所属：岡山大学大学院医歯薬学総合研究科社会環境生命科学専攻総合社会医科学講座

予防歯科学分野　　　　　　　　職名：助教　　氏名：水谷慎介

所属：岡山大学病院予防歯科　　　　　職名：医員　氏名：町田達哉

所属：岡山大学病院予防歯科　　　　　職名：医員　氏名：米田俊樹

所属：岡山大学病院予防歯科　　　　　　　　　職名：医員　　氏名：片岡広太

所属：岡山大学病院予防歯科　　　　　　　　　職名：医員　　氏名：川端勇也

所属：岡山大学病院予防歯科　　　　　　　　　職名：医員　　氏名：山根真由

所属：岡山大学病院予防歯科　　　　　　　　　職名：医員　　氏名：水野裕文

所属：岡山大学病院予防歯科　　　　　　　　　職名：医員　　氏名：國友宗義

所属：岡山大学病院予防歯科　　　　　　　　　職名：医員　　氏名：谷口綾乃

所属：岡山大学病院予防歯科　　　　　　　　　職名：医員　　氏名：宮井久敬

所属：岡山大学病院総合歯科　　　　　　　　　職名：研修医　氏名：内田瑤子

所属：岡山大学病院総合歯科　　　　　　　　　職名：研修医　氏名：福原大樹

所属：岡山大学大学院医歯薬学総合研究科社会環境生命科学専攻総合社会医科学講座

予防歯科学分野　　　　　　　　大学院生　　氏名：杉浦嘉雄

（○：研究責任者）

２４．研究資金および利益相反

本研究は、研究責任者が所属する診療科の厚生科学研究費で実施する。

なお、本研究において、研究者個人の利益・立場等が研究の公平・公正な計画・実施・報告に影響を及ぼすことはない（利益相反はない）。

２５．試料の保存

(1) 試料の保存及び使用方法

研究期間中得られた血液サンプルは血清分離し、再検査が必要な場合に対応するため医局の鍵のかかる部屋にある-80℃冷凍庫に保存する。匿名化された診療情報は、研究責任者のパソコンに保存され、研究終了後に統計解析を行う。

(2) 保存期間

診療情報は、データ解析され研究終了後５年間保存する。また、検体は研究終了後に測定を行った後、匿名のまま廃棄する。

２６．実施計画書等の変更

実施計画書や同意説明文書の変更（改訂）を行う場合は予め臨床研究審査委員会の承認を必要とする。

２７．参考資料・文献リスト

Kiran M, Arpak N, Unsal E, Erdoğan MF. The effect of improved periodontal health on metabolic control in type 2 diabetes mellitus. J Clin Periodontol. 2005; 32: 266-272.
