# Supplementary material for: The effects of non-surgical periodontal treatment on glycemic control, oxidative stress balance and quality of life in patients with type 2 diabetes: A randomized clinical trial
Source: PLoS One. 2017 Nov 16;12(11):e0188171. doi: 10.1371/journal.pone.0188171 (PMC5689834; doi:10.1371/journal.pone.0188171)
Supplement: S2 Protocol — (DOC) [file pone.0188171.s006.doc]

**S2 Protocol. Protocol (in English).**

Clinical study protocol

Clinical study title: Comparison of the effects of oral hygiene instruction and periodontal treatment in patients with type 2 diabetes mellitus
（Comparison of the effects of oral hygiene instruction and periodontal treatment on patients with type 2 diabetes mellitus）

Principal investigator: Manabu Morita

Department of Preventive Dentistry, Okayama University Graduate School of Medicine, Dentistry and Pharmaceutical Sciences, Okayama, Japan

2-5-1 Shikata-cho, Kita-ku, Okayama, 700-8558, Japan

Telephone no. (ext.): 086-235-6710 (6710)

Fax no.: 086-235-6714

E-mail: mmorita@md.okayama-u.ac.jp

Emergency contact (medical office number): 086-235-6712

Planned duration of study: March 1, 2014 – March 31, 2016.

Comparison of the effects of oral hygiene instruction and periodontal treatment in

patients with type 2 diabetes mellitus

Study protocol

1. Background

The “Basic Matters Related to Dental and Oral Health Promotion” are based on the “Dental and Oral Health Promotion Law” enacted in August 2012. It describes the relationship between oral status and general health, between dental diseases and lifestyle habits, and between oral health and medical expenses, as well as the promotion of research on prevention and treatment methods that are effective from their relation to dentistry. Innovation is needed especially in dental-medical collaborative model projects for diabetes patients, as the close relationship between periodontal disease and diabetes continues to be elucidated.

2. Study objectives and necessity

In this study, type 2 diabetes patients being treated on an outpatient basis at Okayama University Hospital will be divided into two groups, an oral hygiene health instruction group (given guidance on plaque control only) and a non-surgical periodontal treatment group (given guidance on plaque control and periodontal treatment). The purpose of this study is to compare the results of tests (clinical and blood biochemistry tests) performed 6 months after the conclusion of periodontal treatment (including full mouth scaling and root planing), and the effects on changes in quality of life (QOL) in these two groups.

The current status of medical-dental collaboration for diabetes patients consists mainly of cooperation between medical and dental institutions by referring patients to each other, and the clinical significance is unclear. The results of this study will give clinical significance to the content of such collaboration (providing support for the improvement in diabetes status from periodontal treatment). This is promising for use as evidence for the introduction of periodontal treatment into health care services covered by health insurance, such as “Dental support additions to diabetes and other patients (provisional)” in future Health, Labour and Welfare administrations.

3. Summary of pharmaceuticals and medical equipment

Not applicable.

4. Subjects

(1) Inclusion criteria:

Patients who meet all of the following criteria will be included as subjects:

1. Currently receiving treatment for type 2 diabetes
2. Capable of participating in the study for 6 months
3. Supported by medical insurance
4. 30 years or older at the onset of study (at the time of informed consent)
5. Consenting patients (Patients who voluntarily provide written consent after receiving a full explanation and fully understanding participation in the study)
6. Outpatients

(2) Exclusion criteria:

Patients who are incompatible for any of the following reasons will be excluded:

- - - - 1. Undergoing dental treatment
        2. Pregnant or possibility of being pregnant
        3. Determined as unsuitable subjects (Other patients judged by the principal investigator or a co-investigator to be unsuitable as subjects)

5. Explanation of study to subjects and obtaining consent

Patients will be given an informed consent form approved by the Ethics Committee, Okayama University Graduate School of Medicine, Dentistry and Pharmaceutical Sciences and Okayama University Hospital, and the study will be fully explained both orally and in writing. Written consent will then be obtained at the discretion of the patients. In the event of a change in the protocol or other matters that may affect consent, such information will be provided to patients promptly and the decision on whether to participate in the study will be confirmed again. At the same time, the informed consent form or other relevant forms will be revised with advance approval by the same Ethics Committee, and consent will again be obtained from patients.

6. Study methods

The following are provided:

(1) Study type/design

Randomized controlled study

(2) Study outline

| **Assessment**  • Primary endpoint: HbA1c  • Secondary endpoints: periodontal status, blood sugar, oxidative stress, and QOL  **Oral hygiene instruction group**  Receives oral hygiene instruction  Recruitment of study participants  **Non-surgical periodontal treatment group**  Receives oral hygiene instruction + scaling and root planning, professional toothbrushing and mechanical tooth cleaning |
| --- |

Figure. Study flow chart

Type 2 diabetes patients in the Nephrology, Diabetology and Endocrinology Department of Okayama University Hospital who consent to participate in the study will be introduced to the Department of Preventative Dentistry outpatient clinic. One member of the research group will separate patients into two groups, the oral hygiene instruction group and the non-surgical periodontal treatment group, using a table of random numbers (the patients will be asked not to inform the examiners of the group to which they are assigned). Thereafter, two dentists blinded to the group assignments will conduct periodontal examinations. Other dentists will then be in charge of oral hygiene instruction and periodontal treatment. For the periodontal examination, calibration will be repeated in advance until the kappa value (a reliability assessment index) exceeds 0.8 for inter- and intra-operator agreement.

The periodontal examination includes probing pocket depth, clinical attachment level (distance from the border between the tooth crown and root to the bottom of the periodontal pocket), bleeding on probing (whether bleeding is seen when the periodontal pocket depth is measured), and the degree of plaque buildup.

Blood tests are routinely conducted as part of regular medical examination in the Nephrology, Diabetology and Endocrinology Department (and will be arranged so that examinations are conducted in the Nephrology, Diabetology and Endocrinology Department and the Department of Preventive Dentistry on the same day). In blood tests, glycated hemoglobin (HbA1c) and glycated albumin will be assessed. Remnant blood from examinations (blood that would otherwise be disposed of as medical waste) will be used by the research group for measurement of the oxidative stress balance.

Some of the above examinations will be performed a total of three times: at the start of the study, after 3 months, and after 6 months. Periodontal treatment will be completed by 2 months from the start of the study. Oral hygiene instruction will be provided to both groups at the start of the study, after 3 months, and after 6 months. In cases where there is a change in diabetes treatment during the study period, the details will be recorded.

(3) Planned duration of subject participation

Six months.

(4) Usage, dosage and administration period of study drugs

Not applicable.

(5) Study drug dosage form/content, properties, packaging, labeling, and storage

Not applicable.

(6) Provisions regarding concomitant medication (therapy)

Not applicable.

(7) Drug withdrawal method

Not applicable.

(8) Management and delivery procedures for study drugs

Not applicable.

(9) Drug administration guidance information

Not applicable.

(10) Case enrollment, allocation

Subject enrollment:

The principal investigator or a co-investigator will: 1) obtain written consent; 2) write the date on which consent was obtained and the items needed to link the subjects to the subject identification code on the subject identification code list kept by the principal investigator; 3) submit the case report form using the subject identification code to the study office (medical office for the Department of Preventive Dentistry); and 4) report immediately in the event of consent withdrawal, discontinuation, or dropout.

Subject allocation:

The subjects will be allocated to the treatment groups with a table of random numbers. Using a stratified block randomization allocation method, adjustments will be made according to baseline serum HbA1c (≥8% and <8%), whether a patient is receiving insulin treatment, and types and number of drugs (≤2 or ≥3) a patient is taking.

(11) Handling at completion of study

After the completion of the study, the principal investigator will provide the treatment he considers to be the most appropriate for each subject, including treatment based on the findings obtained in this study.

7. Assessments

(1) Primary endpoint

Serum HbA1c (National Glycohemoglobin Standardization Program [NGSP] value)

(2) Secondary endpoints

Periodontal status (number of teeth present, probing pocket depth, clinical attachment level, bleeding on probing, Plaque Control Record)

Hematological index (glycated albumin)

Blood biochemical index (oxidative stress balance)

QOL

(3) Safety assessment

Not applicable.

8. Observation and test items

(1) Patient characteristics: Chart number, patient’s initials, sex, date of birth, race, inpatient or outpatient, height, weight, complications, past medical history, history of present illness, previous treatment, and other.

(2) Confirmation of subjective and objective symptoms: Confirmation by interview (with reference to the symptom diary if available).

(3) Blood test: Same test items as in regular medical examination.

(4) Blood biochemistry test: Same test items as in regular medical examination. Factors associated with oxidative stress will also be assessed to confirm the relationship between oral health and whole body status.

(5) Periodontal examination: Number of teeth present, probing pocket depth, clinical attachment level, bleeding on probing, and Plaque Control Record.

(6) Questionnaire survey on QOL.

Table. Study Schedule

| Item | | Study initiation | Period of periodontal treatment | Observation period | |
| --- | --- | --- | --- | --- | --- |
| Timing | | Week 0 | Less than 2 months | After 3 months | After 6 months |
| Examination | | Examination 1 | Examination 2–5 | Examination 6 | Examination 7 |
| Consent obtained | | ○ |  |  |  |
| Confirmation of patient characteristics^a^ | | ○ |  |  |  |
| Oral hygiene instruction | |  |  |  |  |
| Periodontal treatment | |  |  |  |  |
| Confirmation of subjective and objective symptoms | | ○ |  | ● | ● |
| Questionnaire survey on QOL | | ○ |  | ● | ● |
| Clinical tests | Blood tests^b^ | ○ |  | ● | ● |
|  | Blood biochemistry  tests^c^ | ○ |  | ● | ● |
| Periodontal examination | | ○ |  | ● | ● |

Open circles (○) indicate measures that will be performed prior to dental intervention. Black circles (●) indicate measures that will be performed after dental intervention.

^a^Patient characteristics indicate characteristics routinely noted in medical care, such as age, sex, past medical history, and complications.

^b^Blood tests indicate the same tests performed during regular medical examination.

^c^Blood biochemistry tests indicate the same tests performed during regular medical examination. In addition, factors associated with oxidative stress will be assessed to confirm the relationship between oral health and whole body status.

9. Discontinuation criteria

- 1. A subject asks to stop participating in the study or withdraws consent.
  2. It is found after enrollment that eligibility requirements are not fulfilled.
  3. Continuation in the study is difficult due to exacerbation of complications.
  4. A subject is discovered to be pregnant.
  5. The entire study is discontinued.
  6. Dental treatment is found to be necessary for a subject in the oral hygiene instruction group after assignment.
  7. A physician determines that discontinuation is advisable for some other reason.

10. Handling when an adverse event occurs

(1) Response to patients when an adverse event occurs

When an adverse event that requires treatment occurs in a subject, the subject will be notified and appropriate medical care for the adverse event will be provided.

(2) Report of a serious adverse event

If a serious adverse event occurs or there is concern of such an event occurring, a report will be made immediately to the Hospital Director of Okayama University Hospital.

11. Report of deviation from protocol

The following matters are provided in accordance with GCP:

- The principal investigator and co-investigators will not deviate from or make changes to the study protocol before obtaining approval from the Hospital Director based on advance review of the Ethics Committee.
- The principal investigator and co-investigators may deviate from or change the study protocol before obtaining advance approval from the Ethics Committee for compelling reasons in order to avoid an emergency. In such cases, the principal investigator or a co-investigator will submit the details regarding the deviation or change and the associated reasons, together with a proposal for revision of the study protocol if necessary, to the Ethics Committee without delay, and approval from the Ethics Committee and Hospital Director will be obtained.
- In cases of deviation from the study protocol, the principal investigator or a co-investigator will record all items of deviation and the reasons for them.

12. Completion, discontinuation, or cessation of study

(1) Study completion

At the completion of the study, the principal investigator will submit a study completion report to the Hospital Director without delay.

(2) Study discontinuation or cessation

The principal investigator will consider whether or not to continue the study in the event of either of the following:

1) Recruiting subjects is difficult and reaching the planned number of subjects is judged to be unlikely.

2) The Ethics Committee asks for changes to be made in the protocol or other matters, and those changes are judged to be difficult to make.

When a decision is made to discontinue or cease the study, a report to that effect together with the reason will be made in writing to the Hospital Director without delay.

13. Study period

From March 1, 2014 to March 31, 2016.

14. Data aggregation and statistical analysis

Data analysis will be conducted from the following observations:

1) Role of plaque control instruction in type 2 diabetes patients: The significance of plaque control instruction will be investigated from a comparison between the start of the study and the time of reassessment in the oral hygiene instruction group, including its effect on QOL.

2) Significance of periodontal treatment: The effects of periodontal treatment in type 2 diabetes patients will be discerned from a comparison of the oral hygiene instruction group and the non-surgical periodontal treatment group.

15. Target patient number and rationale

The target patient number is 20 for each group (40 patients total).

The number of patients is calculated to be 20 based on analysis of the serum HbA1c level in a previous study (see reference) on periodontal treatment in diabetes patients. To determine the sample size when the *t*-test is used, calculations were performed using SPSS Samplepower (IBM, Tokyo, Japan). Under the conditions of α=0.05 and β=0.20, with a two-tailed *t*-test, the predicted HbA1c level was 7.31% in the oral hygiene instruction group and 6.51% in the non-surgical periodontal treatment group. With an assumed standard deviation of 0.8, significance was hypothesized when the mean serum HbA1c differed by ≥0.8% as a result of periodontal treatment. As a result, 17 patients are needed for the study. Assuming a 15% dropout rate, the number is set at 20 patients.

16. Subjects’ rights, safety, and disadvantage

(1) Human rights (protection of privacy)

Subject data are coded and test results are provided to the person conducting the analysis at Okayama University so that individual subjects cannot be identified. To combine the data from the examination table, questionnaire, and each test, the same symbols are given to the same subject being examined. After the study has been completed, these data will be destroyed.

(2) Patient safety, disadvantage

Health insurance and other measures needed to compensate for health damage to subjects are not adopted.

Reason: There is the possibility only of slight pain when blood is taken. No damage to health is foreseen.

17. Cost to patients

Within the range of regular medical examination.

18. Expected benefit from participating in clinical study, and possible risks

A benefit from participating in this study is that subjects are given a 2,000 yen QUO card as remuneration each time they undergo medical examinations. A disadvantage is that each test takes time. There is no risk.

19. Compensation for damage to health

Health insurance and other measures needed to compensate for health damage to subjects are not adopted.

Reason: This study is performed within the range of regular medical examination, and no damage to the subjects’ health is foreseen.

20. Correspondence with GCP and Helsinki Declaration

This study will be conducted in accordance with GCP and adhere to the principles laid down in the Helsinki Declaration (2008 revision).

21. Storage of records

The principle investigator will keep all required documents related to conducting this study (copies of application forms, correspondence from Hospital Director, copies of all applications and reports, subject identification code list, consent forms, copies of case reports, and other documents or records needed to guarantee the reliability of data) and dispose of them after the study has been published.

22. Publication of study results

The study results will be compiled in a report by April 30, 2016, and reported to the Hospital Director. The study results will also be used in journal publication(s) and conference presentation(s). When the study results are made public, personal information that could be used to identify individual subjects will not be disclosed.

23. Research organization

Affiliation: Department of Preventive Dentistry, Okayama University Graduate School of Medicine, Dentistry and Pharmaceutical Sciences

Title: Professor

Name: Manabu Morita○

Affiliation: Department of Public Health, Okayama University Graduate School of Medicine, Dentistry and Pharmaceutical Sciences

Title: Professor

Name: Keiki Ogino

Affiliation: Department of Nephrology, Rheumatology, Endocrinology, and Metabolism, Okayama University Graduate School of Medicine, Dentistry, and Pharmaceutical Sciences

Title: Associate Professor

Name: Jun Wada

Affiliation: Preventive Dentistry, Okayama University Hospital

Title: Lecturer

Name: Takaaki Tomofuji

Affiliation: Preventive Dentistry, Okayama University Hospital

Title: Lecturer

Name: Daisuke Ekuni

Affiliation: Department of Preventive Dentistry, Okayama University Graduate School of Medicine, Dentistry and Pharmaceutical Sciences

Title: Assistant Professor

Name: Tetsuji Azuma

Affiliation: Center for Innovative Clinical Medicine, Okayama University Hospital

Title: Assistant Professor

Name: Takayuki Maruyama

Affiliation: Department of Preventive Dentistry, Okayama University Graduate School of Medicine, Dentistry and Pharmaceutical Sciences

Title: Assistant Professor

Name: Shinsuke Mizutani

Affiliation: Preventive Dentistry, Okayama University Hospital

Title: Physician

Name: Tatsuya Machida

Affiliation: Preventive Dentistry, Okayama University Hospital

Title: Physician

Name: Toshiki Yoneda

Affiliation: Preventive Dentistry, Okayama University Hospital

Title: Physician

Name: Kota Kataoka

Affiliation: Preventive Dentistry, Okayama University Hospital

Title: Physician

Name: Yuya Kawabata

Affiliation: Preventive Dentistry, Okayama University Hospital

Title: Physician

Name: Mayu Yamane

Affiliation: Preventive Dentistry, Okayama University Hospital

Title: Physician

Name: Hirofumi Mizuno

Affiliation: Preventive Dentistry, Okayama University Hospital

Title: Physician

Name: Muneyoshi Kunitomo

Affiliation: Preventive Dentistry, Okayama University Hospital

Title: Physician

Name: Ayano Taniguchi

Affiliation: Preventive Dentistry, Okayama University Hospital

Title: Physician

Name: Hisataka Miyai

Affiliation: Preventive Dentistry, Okayama University Hospital

Title: Resident

Name: Yoko Uchida

Affiliation: Preventive Dentistry, Okayama University Hospital

Title: Resident

Name: Daiki Fukuhara

Affiliation: Department of Preventive Dentistry, Okayama University Graduate School of Medicine, Dentistry and Pharmaceutical Sciences

Post Graduate student

Name: Yoshio Sugiura

(○: Principal investigator)

24. Grant and conflict of interest

This study will be conducted with funding from a grant from the Ministry of Health, Labour and Welfare.

In this study, the researchers’ individual benefit or standing has no effect on fair and impartial planning, implementation, and reporting of the study (there are no conflicts of interest).

25. Storage of samples

(1) Methods of storing and using samples

Sera will be separated from blood samples obtained during the course of this study and stored in a freezer at −80°C in a locked room in the medical office for use in the event that retesting is necessary. Anonymized treatment information will be kept on the principal researcher’s computer and statistically analyzed at the completion of the study.

(2) Storage duration

Treatment information will be kept for 5 years after the data are analyzed and the study is completed. Specimens measured after the completion of the study will be disposed of after the measurements, maintaining anonymity.

26. Changes to protocol

Changes (revisions) to the protocol or informed consent forms require advance approval of the Ethics Committee, Okayama University Graduate School of Medicine, Dentistry and Pharmaceutical Sciences and Okayama University Hospital.

27. Reference materials/reference list

Kiran M, Arpak N, Unsal E, Erdoğan MF. The effect of improved periodontal health on metabolic control in type 2 diabetes mellitus. J Clin Periodontol. 2005; 32: 266-272.
